# *Aeromonas* wound infection in a healthy boy

**DOI:** 10.1099/jmmcr.0.005129

**Published:** 2017-11-14

**Authors:** Bart Rutteman

**Affiliations:** Department of Pediatrics, AZ Sint Blasius Dendermonde, 9200 Dendermonde, Belgium

**Keywords:** *Aeromonas*, wound infection, fluoroquinolone

## Case summary

A 12-year-old boy let go too soon from a rope swing over water, and fell onto rocks with his knees. In the emergency ward (AZ Sint Blasius, Dendermonde, Belgium), he was diagnosed with a penetrating wound of the left lower leg, exposing a part of the proximal tibia. The wound was sutured subcutaneously and cutaneously, and he was given amoxicillin/clavulanic acid (875/125 mg twice daily).

The day afterwards (day 1), the boy returned to the emergency ward with a fever (38.5 °C). Physical examination showed no signs of sepsis, a normal ear-nose-throat inspection, and the stitched wound had a normal aspect. Laboratory investigations showed a slight leucocytosis (12.600 cells µl^−1^) with neutrophilia (85 %), but a normal C-reactive protein level (6.6 mg l^−1^) and sedimentation rate (2 mm h^−1^). He was hospitalized andamoxicillin/clavulanic acid was continued (dosed 1000/10 mg kg^−1^ in 4 doses).

The boy remained febrile (up to 38.9 °C) on day 2. Examination now showed evacuation of some pus from his leg wound, with a slight swelling, redness and local pain. The pus was sent for culture. Ultrasound of the leg showed a small (11×3 mm) subcutaneous collection. Bone scintigraphy showed increased blood flow in the cranial part of the left lower leg with global swelling, indicating an important soft tissue inflammation.

Cultures of the pus showed growth of *Aeromonas* species (on both samples taken), being resistant to amoxicillin/clavulanic acid. The finding of *Aeromonas* species came unexpectedly. Only after he was actively asked did the patient admit to having fallen into the ditchwater (stagnant river) after having injured his knee. Taking this into account, the *Aeromonas* species was considered to be the pathogen of his wound infection. Therefore, antibiotic treatment was switched to ciprofloxacin (30 mg kg^−1^ in 2 doses) based on the antibiogram. Thereafter, the boy showed a swift healing of the wound.

**Question**

Which information is most helpful to determining the choice of empiric antibiotic therapy for infectious wounds?

**Answer options**

1. The location of the wound

2. A thorough history including trauma mechanism and circumstances

3. The presence or absence of pus

4. The presence of absence of fever

## Discussion

**Correct answer:** 2. A thorough history including trauma mechanism and circumstances.

Wounds that have been exposed to (stagnant) water and/or dirt can get infected with *Aeromonas* species, which are almost synonymous with water and aquatic environments [1]. *Aeromonas* species are practically always resistant to penicillins (including those with β-lactamase coverage) and should be treated with fluoroquinolones [1–6].

It is, therefore, of great importance to ask for the trauma mechanism and circumstances in which the wound occurred. When there is a history of water and/or dirt exposure, empiric therapy should include a fluoroquinolone.
